# Cardiovascular Risk and Statin Therapy Considerations in Women

**DOI:** 10.3390/diagnostics10070483

**Published:** 2020-07-16

**Authors:** Gina Gheorghe, Peter P. Toth, Simona Bungau, Tapan Behl, Madalina Ilie, Anca Pantea Stoian, Ovidiu Gabriel Bratu, Nicolae Bacalbasa, Marius Rus, Camelia Cristina Diaconu

**Affiliations:** 1 Department 5, “Carol Davila” University of Medicine and Pharmacy, 050474 Bucharest, Romania; gheorghe_gina2000@yahoo.com (G.G.); drmadalina@gmail.com (M.I.); 2 Ciccarone Center for the Prevention of Cardiovascular Disease, Johns Hopkins University School of Medicine, Baltimore, MD 21205, USA; peter.toth@cghmc.com; 3 Department of Pharmacy, Faculty of Medicine and Pharmacy, University of Oradea, 410028 Oradea, Romania; 4 Chitkara College of Pharmacy, Chitkara University, Punjab 140401, India; tapanbehl31@gmail.com; 5 Gastroenterology Department, Clinical Emergency Hospital of Bucharest, 105402 Bucharest, Romania; 6 Department 2, “Carol Davila” University of Medicine and Pharmacy, 050474 Bucharest, Romania; ancastoian@yahoo.com; 7 Department 3, “Carol Davila” University of Medicine and Pharmacy, 050474 Bucharest, Romania; ovi78doc@yahoo.com; 8 Department of Surgery, “Ion Cantacuzino” Clinical Hospital, 030167 Bucharest, Romania; nicolae_bacalbasa@yahoo.ro; 9 Department 13, “Carol Davila” University of Medicine and Pharmacy, 050474 Bucharest, Romania; 10 Department of Medical Disciplines, Faculty of Medicine and Pharmacy, University of Oradea, 410073 Oradea, Romania; rusmariusr@yahoo.com; 11 Internal Medicine Department, Clinical Emergency Hospital of Bucharest, 105402 Bucharest, Romania

**Keywords:** cardiovascular risk factors, dyslipidemia, myopathy, statin, women

## Abstract

Despite major progress in the prevention and treatment of cardiovascular diseases, women remain an underdiagnosed and insufficiently treated group, with higher hospitalization and death rates compared to men. Obesity, more frequently encountered in women, raises the risk of metabolic syndrome and cardiovascular diseases as women age. There are some differences based on sex regarding the screening, diagnosis, and treatment of dyslipidemia, as it has been observed that women are less frequently prescribed statins and, when they are, they receive lower doses, even after myocardial infarction or coronary revascularization. Real-life data show that, compared to men, women are at higher risk of non-adherence to statin treatment and are more predisposed to discontinue treatment because of side effects. Statin metabolism has some particularities in women, due to a lower glomerular filtration rate, higher body fat percentage, and overall faster statin metabolism. In women of fertile age, before initiating statin treatment, contraception methods should be discussed because statins may have teratogenic effects. Older women have a higher likelihood of polypharmacy, with greater potential for drug interactions when prescribing a statin.

## 1. Introduction

Cardiovascular diseases (CVDs) have a considerable impact both on public health and global economy because of their substantial costs. Studies published in the scientific literature reported a direct cause–effect relationship between cardiovascular risk factors and certain clinical and preclinical conditions, such as premature mortality, cardiovascular mortality, arterial stiffness, heart failure (HF), stroke, decline in cognitive function, depression, venous thromboembolism, pulmonary emphysema, chronic obstructive pulmonary disease (COPD), and renal disease [1,2]. The main modifiable cardiovascular risk factors are smoking, obesity, metabolic syndrome, sedentary lifestyle, high levels of atherogenic lipoproteins and triglycerides (TGs), high blood pressure, diabetes mellitus (DM), and chronic renal disease [2]. These risk factors are interrelated. In this review, we focus on dyslipidemia and lipid-lowering therapy in women.

### 1.1. Smoking

In 2015, in the US, smoking prevalence was estimated at 15.1% among adults and 4.2% in teenagers [3]. In 2018, approximately 13.7% (34.2 million) of US adults were current cigarette smokers, and most of them (74.6%) smoked daily [4]. At the international level, tobacco consumption represents the main preventable cause of death. In 2015, smoking was estimated to be responsible for 7.2 million deaths worldwide [1]. Moreover, it increases the risk both for stroke and ischemic heart disease, with it being recognized that regarding cardiovascular events, smoking is considered a relevant risk factor. While in the case of stroke, the risk is similar for men and women, in the case of ischemic heart disease the risk is 25% higher in smoking women compared to men [2]. Cigarette smoking was proven to enhance the risk of atherosclerosis by lowering high-density lipoprotein cholesterol (HDL-C) levels, TGs, and potentiating insulin resistance [5]. Results of clinical trials reported the beneficial effects of treatment with lipid-lowering drugs (particularly statins) in dyslipidemic patients [6].

Few studies were focused on the gender difference concerning the cardiovascular risk derived from smoking and interference with CVD risk reduction due to statin treatment. There are published clinical pieces of evidence that smokers (as a subgroup) are the ones who benefit most from the effects of statin treatment. Considering that smoking is more common in men vs. women, obviously there is a higher use of statins in men vs. women. The study by Zhang et al. included multivariable analyses of 24,338 patients (representing 90.4% of the gender difference in persistent statin therapy, *p* < 0.0001 for all), who met four inclusion criteria, as follows: Smoking history, evaluation by a cardiologist, younger age, and no report of adverse reactions to statins; these subjects were more likely to have persistent statin therapy [7].

Another meta-analysis suggested that the effect of statin treatment on CVD is similar in smokers and non-smokers. Regarding the number of avoided cardiovascular events, smokers have no significant benefit from the effects of statins than non-smokers [8].

Although statins reduce CVD morbidity and mortality in current smokers with CVD, the best results were obtained for non-smokers treated with drugs of this class, as follows: For primary prevention, pravastatin or lovastatin; and for secondary prevention, simvastatin or pravastatin. Smokers on placebo had the highest risk of such events, no matter the group. Correct and detailed knowledge (based on these data) of both clinicians and patients can lead to a better awareness of the beneficial outcome of effective treatment (such as the use of statins), as well as regarding the harmful effects of smoking [9].

### 1.2. Sedentary Lifestyle

Only 27.1% of teenagers are compliant with recommendations regarding daily physical activity, a percentage comparable to that in adults [2]. In 2015, compared to 2009, the proportion of young people who spend more than 3 hours a day in front of the computer rose from 24.9% to 41.7% [2]. Recent data about physical activity in adults from the US reveal that more than 25% spend over 8 hours/day sitting, 4/10 are physically inactive, and 1 in 10 report both. Sedentary behavior can lead to obesity, high blood pressure, and dyslipidemia. It was shown that physical activity could improve these factors [10].

These worrisome percentages explain the decrease of age at the time of first heart attack or stroke but also the increase in the number of such events [2]. Physical training is inversely correlated to all-cause mortality, even among dyslipidemic patients who do not receive statin therapy, and this is significant especially for patients with contraindications for this treatment (such as women of fertile age) or who refuse it despite the recommendations [11]. Dyslipidemic patients who perform physical training and follow statin therapy benefit most, because the two are independently associated with lower mortality [12]. Moreover, individuals who follow a combined therapy with statin and exercise have better outcomes compared to statin monotherapy in terms of insulin sensitivity, inflammation, and exercise capacity [13].

Physical activity has different effects, depending on the type of lipoprotein. One meta-analysis of 25 randomized controlled trials showed that physical training alone, at an equivalent of 5.3 metabolic equivalents of tasks (METs), improved HDL-C by 2.53 mg/dL [14]. The impact on low-density lipoprotein cholesterol (LDL-C) is modest, and it is noticeable if associated with bodyweight loss; for each dropped kilogram, LDL-C decreases by 0.8 mg/dL [15]. The effect of fitness training on TG values is still a matter of debate. There are studies that found significantly lower values [16] of TGs after only two days of exercise and others that did not find any difference after training sessions [17]. However, if a patient is significantly insulin resistant and has hypertriglyceridemia, then weight loss typically reduces insulin resistance and leads to improved clearance of TGs secondary to decreased apoprotein CIII and increased activity of lipoprotein lipase [18]. 

Treatment with statins, complementary to physical activity, may subject the patients to a higher risk of myopathy, especially in those who receive high doses of the drug and have intense physical activity, as well as seniors. The recommended exercises and an optimal physical activity program must precede the statin prescription and treatment [19]. So far, Bosomworth’s study is considered the first randomized proof study that confirms the negative outcome of statin treatment on energy consumption and patient exhaustion. These results were described in a somewhat healthy group of patients, who were administered low doses of statins, and in whom both pravastatin and simvastatin led to considerable adverse effects on energy and fatigue. Women were extremely vulnerable to these side effects; in the literature, cases are reported that highlight this type of adverse action. Findings and data provided are important in terms of assessing the energetic and functional state, as well as the general well-being of the patient [20].

### 1.3. Obesity

According to the National Health and Nutrition Examination Survey (NHANES), between 2011–2012 and 2013–2014, obesity prevalence rose from 34.9% to 37.7% in adults and from 16.9% to 17.2% in teenagers. Likewise, a higher prevalence of obesity was found in women (34.4% for ages 20–39 years and 42.1% in the 40–59 years age group) than in men (30.3% for ages 20–39 years and 38.3% in the 40–59 years age group) [21]. A 2016 meta-analysis highlighted an increase in the cardiovascular risk of obese individuals without metabolic syndrome, compared to individuals with normal weight, suggesting that obesity is a risk factor, even in the absence of high blood pressure, DM, or elevated cholesterol levels [2]. Dyslipidemia is an important comorbidity of obese patients, being characterized by high fasting TGs, low HDL-C, and normal LDL-C and total cholesterol (TC) [22]. Diet and physical activity in patients with metabolic syndrome decrease TG levels and increase HDL-C levels, without significant effects on TC and LDL-C [23]. Dyslipidemia in severely obese subjects is related to weight distribution, insulin sensitivity, and impaired glucose tolerance [22,24].

Numerous epidemiological studies have been conducted mainly on male patients, as CVDs have been regarded as “male diseases” for a long time, due to an earlier onset in men vs. women; this fact determined fewer efforts to prevent and treat CVDs in women. Moreover, obese European women seem to be at increased risk of psychological dysfunction compared to obese men, which can be explained by the greater social pressure on European women to be slim. As recent research has shown, obese women are likely to develop left ventricular concentric/eccentric hypertrophy, while obese men predominantly develop concentric hypertrophy (which is responsible for cardiovascular mortality more than eccentric hypertrophy) [25].

### 1.4. Metabolic Syndrome

It represents a series of changes induced by insulin resistance. Insulin resistance is associated with an increased waist circumference and visceral adiposity, hyperglycemia, elevated blood pressure, reductions in HDL-C, elevations in TG, and increased small dense LDL particles [26,27]. These changes augment the risk of CVD. It is estimated that 47 million US individuals have metabolic syndrome, which increases their risk of coronary heart disease and stroke threefold. The prevalence of metabolic syndrome increases with age from 7% at the age of 20–29-year-old to 44% in 60–69-year-olds. The prevalence is increasing amongst children and adolescents [28]. According to NHANES data from 2001 to 2012, the prevalence of metabolic syndrome is higher in women (34.4%) compared to men (29%). The risk of metabolic syndrome increases with age, as individuals undergo progressive weight gain [2].

### 1.5. High Levels of Cholesterol and Total Lipids

Dyslipidemia may be defined as high levels of serum TC, LDL-C, and TG, or a decreased serum HDL-C concentration [29]. Serum TG, TC, LDL-C, HDL-C, TC/HDL-C, and LDL-C/HDL-C ratios are independent predictors of CVD risk [30]. While in adults over 20 years old, in the US, the prevalence of hypercholesterolemia is 11.9%, 48.6% of adults over 40 years old are candidates for statin therapy, according to the American College of Cardiology/AHA guidelines for the management of blood cholesterol [2]. Currently, the main objective of the management of dyslipidemia is to lower serum LDL-C levels [31]. Meta-analyses confirmed that atherosclerotic CVD reduction is dose dependent with LDL-C-lowering agents, and the higher the LDL-C reduction, the greater the CV risk reduction [32]. The advantages of reducing LDL-C are not specific for statin treatment, and there is not a cut-off that can be considered too low and thus harmful [33].

In the premenopausal period, a less proatherogenic plasma lipid profile is observed in women compared to men of the same age; in other words, women have a higher concentration of HDL-C and lower concentrations of very low-density lipoprotein cholesterol (VLDL), LDL-C, total plasma TG, and VLDL-TG concentrations (both under normal diet and fasting conditions) [34].

There is a traditional outlook that the female "advantage" in pre-menopause is given by the cardioprotective lipid profile; this is due to differences in sex hormones in men and women, especially the availability of estrogens and androgens. According to studies on the effect of normal physiological variations in the hormonal environment (during the menstrual cycle or at menopause), they are no longer entirely favorable to women, especially when the actions of exogenous sex steroid intake on plasma lipids concentrations and kinetics are considered [34,35]. The onset of menopause associated with the loss of ovarian hormones often comes with changes in the relative distribution of body fat, from a subcutaneous to a more visceral distribution [36,37].

Compared to men, women have their first heart attack almost 10 years later; this fact can be determined to a considerable extent, before menopause, by the actions and effects of estrogen on cholesterol metabolism [38].

Moreover, after the onset of menopause and decreased levels of ovarian hormones, women are less likely to develop CVD than men. The prevalence of coronary heart disease (CHD) is 19.7% in men vs. 11% in women, aged between 60–79 years, and shows the same trend in the elderly, according to published data [39].

### 1.6. High Blood Pressure

Between the years 2005 and 2015, the rate of deaths attributed to high blood pressure increased by about 10.5%. In total, 874 million adults worldwide had systolic blood pressure greater than 140 mmHg in 2015 [2]. The term dyslipidemic hypertension (DH) or lipitension was used to describe a genetic syndrome found in approximately 12% of the patients with primary high blood pressure and 48% of the hypertensive sibships [40]. The risk of CVD resulting from concomitant high blood pressure and dyslipidemia is more multiplicative than the sum of these individual risk factors [41]. The prevalence of lipitension ranges from 15% to 31% in the US; 31% of the elderly have it, and the incidence is higher in women compared to men (20% vs. 16%) [42]. The prevalence varies with the number of risk factors [43]. Dyslipidemia causes endothelial dysfunction and loss of physiological vasomotor activity, which can lead to hypertension [44]. Cross-sectional and prospective studies highlighted the interplay between abnormal lipids and hypertension [45]. This interaction increases the CVD risk exponentially and a decrease of the prevalence of "lipitension" can significantly ameliorate the outcomes [46].

### 1.7. Diabetes Mellitus

In the year 2015, about 5.2 million deaths worldwide were attributed to DM [2]. Diabetes is highly prevalent throughout the world and accelerates atherogenesis. Diabetes is unequivocally associated with an increased risk of acute cardiovascular events and cardiovascular mortality, as well as microangiopathy, including proliferative retinopathy, neuropathy, and nephropathy. The risk of CVD is enhanced 2-fold in diabetic men and 3–4-fold in diabetic women [47]. Although both types of diabetes enhance the risk of CVD, the more harmful effect is exerted by type 2 diabetes mellitus (T2DM) [48,49].

An aggressive glycemic control is enough to minimize the CV risk of type 1 diabetes mellitus (T1DM) but is insufficient for T2DM [50]. Women with T1DM have a double risk of fatal and nonfatal vascular events compared to men with T1DM [51]. 

The coexistence of renal disease elevates the risk of CVD [52]. The risk of cardiovascular events in patients with diabetes can be reduced through measures that aim to improve the management of dyslipidemia, high blood pressure, and other CV risk factors [53,54].

Studies showed that high LDL-C and non-HDL-C levels and decreased HDL-C levels are associated with a higher CVD risk in patients with diabetes [54]. 

The average lipid profile is different in T1DM and T2DM; in T1DM, if there is good glycemic control, it looks similar to the general population, whereas in T2DM, there are lipid abnormalities, regardless of glycemic control [50,55].

In both types of diabetes, inadequate glycaemic control augments serum TG, VLDL, and intermediate-density lipoprotein (IDL) levels, and lowers HDL-C [56]. In these patients, the effects of glucose-lowering medications on the lipid profile vary greatly; metformin decreases TG and only modestly decreases LDL-C, sulfonylureas have no effect, dipeptidyl peptidase-4 (DPP-4) inhibitors lower postprandial TG, GLP1 analogues minimize both fasting and postprandial TG and enhance HDL-C, and acarbose lowers postprandial TG. Pioglitazone and rosiglitazone lower TG and enhance HDL-C, with an insignificant increase of LDL-C, but a decrease in small dense LDL-C. With gliflozins, sodium-glucose co-transporter-2 (SGLT2), there is a small augmentation in both LDL-C and HDL-C. Insulin has no effect on the lipid profile [57].

DM is characterized by a modified lipid profile, with dyslipidemia determining cardiovascular complications in diabetic patients (most often CHD) [50,55,56]. For diabetic women, the components of the increased relative risk of CHD continue to be insufficiently acknowledged. Nevertheless, the negative effects determined by T2DM on cardiovascular risk factors (like TG, HDL-C, LDL-C) and blood pressure were more accentuated in women than in men. The study of Aderibigbe et al. on diabetic females versus males revealed significantly higher values of LDL-C and TC (*p* < 0.05) in the case of diabetic females. A reduced HDL-C value was also noticed in the case of diabetic females in contrast with diabetic males. These observations are consistent with previously published data [58,59].

### 1.8. Chronic Renal Disease

CVDs are the main cause of death in individuals with renal disease. Between 2005 and 2015, the prevalence of kidney disease increased by 27%, with 323 million people worldwide affected by kidney disease in 2015. In the US, in 2014, approximately 50 billion dollars were spent on the care of patients with renal diseases. From those, more than 70% have been attributed to patients with comorbidities, such as DM and HF [2,60]. It is known that renal disease is associated with disruption in lipoprotein metabolism and serum lipid levels, with increased TG, low HDL-C, and elevations in small dense LDL particles [61].

From a clinical point of view, women’s cardiovascular risk is often misunderstood and misdiagnosed. This can be explained by several factors, such as [62,63]:

- Lack of awareness: CVDs are perceived as a disease of men [62,63].
- Lack of recognition of sex differences: Symptoms are different from those of men and, from an early age, women are socialized to accept discomfort [62,63].
- Women’s health risks are misunderstood; women are more concerned with the risk of cancer, especially breast cancer [62,63]. It has also been observed that the use of traditional cardiovascular risk and the Framingham score underestimates cardiovascular risk in women [62,63]. It is recommended to use unique risk factors for a better estimation (Table 1) [64].

As the United States Renal Data System (USRDS) reveals, 62% of CKD subjects who attained end-stage renal failure in 2015 were males, while hardly 38% were women [64]. Moreover, as stated before, women with CKD present a reduced deterioration of the renal function in time in contrast with men [65,66].

The important gender differences are obvious in the incidence and load of different CVDs, even though CVD is still the major cause of death both in men and women in the US, the main cause of CVD mortality/morbidity remaining CHD. There are more cases of women who live with or die of stroke/CVD than men, as well as more hospital discharges due to stroke and HF [67].

Women dying from stroke in 2007 represented 60.6% of the total number of deaths due to this condition, but, in comparison, the number of men dying of or living with CHD is greater than the number of women and they also have more hospital discharges. Additionally, the increased incidence of CHD in men in every age group until 75 years was highlighted, which may promote the idea that heart disease is characteristic to men. Gender variations in CHD and CVD mortality mostly evidence gender variations in the US demographics. As females have a longer lifespan than males, women represent an important part of the senior population in whom CVD occurrence is more frequent. Research conducted for women in the age group of 35–44-year-olds reveals that CHD mortality increased statistically significantly (on average by 1.3%/year) in the interval 1997–2002 [68].

## 2. Differences between Sexes Regarding Screening, Diagnosis, and Treatment of Dyslipidemia

Some studies have underlined the differences in the screening, diagnosis, and treatment of dyslipidemia in women, a major risk factor with a direct impact on cardiovascular mortality. Thus, despite the high number of cardiovascular-related deaths in women (for example, in 2007, CVDs were responsible for one death/minute in women from the US), only 72% of women undergo screening for dyslipidemia. The Prospective Multicenter Imaging Study for evaluation of Chest Pain (PROMISE) [69] trial highlights that, despite the identical or even higher risk profile of women for CVD, there is a wrong tendency to include them in a lower risk category than men [70,71]. Another fact worth mentioning with a direct impact on morbidity/mortality is that women receive statins more rarely and in lower doses, even after an acute myocardial infarction (MI), although the benefits of statin treatment are similar in men and women [72,73]. Another study published in August 2019 showed that compared to men, the percentage of women with dyslipidemia who receive treatment with statins is smaller (67% vs. 78.4%) [74]. Among women who do receive statins, only 36.7% are given the right dose compared to men (45.2%), with the right dose defined as the dose that lowers LDL-C levels under 100 mg/dL, or under 70 mg/dL in patients with very high cardiovascular risk [74,75]. Furthermore, it was demonstrated that women are more likely to stop treatment with statins in comparison to men (10.9% vs. 6.1%) and to take a lower dose than recommended (3.6% vs. 2%). On the other hand, there are differences between genders regarding the understanding of the cardiovascular risk and the benefits of statins. Although women were more concerned about the risk of stroke or MI compared to men (45.7% vs. 34.4%), they were less aware of the importance of hypercholesterolemia to the CVD (75.4% vs. 82.1%) and of the benefits of statin treatment in lowering cardiovascular risk (68.0% vs. 73.2%) [74]. Another factor that played an important role in women’s low compliance to statin treatment was the fear of adverse reactions, such as muscular symptoms and hepatic cytolysis [74]. 

The Understanding Statin Use in America and Gaps in Patient Education (USAGE) survey [76] compared the risk of non-adherence to statin treatment between men and women, and concluded that women are more exposed to this risk, with the main reasons being the muscular side effects but also the cost of treatment, with women tending to put the needs of their family above their own needs [77,78]. Another factor that may contribute to the dissatisfaction with statin treatment is a flawed doctor–patient relationship [77,78]. Thus, compared to men, the number of women who reported that their physician did not provide them with enough information regarding their CVD, the importance of achieving the lipid objectives, and the benefits of statins was higher [77].

## 3. Statin Metabolism and Peculiarities in Women

Statins fall into two main categories: Fungal derived and synthetically produced. The two categories are differentiated by their ability to interact and inhibit 3-hydroxy-3-methyl-glutaryl-coenzyme A (HMG-CoA) reductase (the rate-limiting step in cholesterol biosynthesis) and by their hydrophobicity (Table 2). Thus, due to their structural characteristics, synthetic statins have a higher affinity to HMG-CoA reductase. The pharmacokinetics of statins vary depending on their solubility. In the case of lipophilic statins, such as lovastatin, simvastatin, Fluvastatin, and atorvastatin, they passively diffuse intracellularly, and the main route of excretion is through the biliary system. In the case of hydrophilic statins, such as rosuvastatin or pravastatin, the intracellular transport is predominantly active, and excretion is done both by the liver and kidneys. The absorption and bioavailability of statins may be affected by food intake. If in the case of atorvastatin, pravastatin, and Fluvastatin, food intake leads to reduced bioavailability, in the case of lovastatin, food intake leads to increased bioavailability. The pharmacokinetic characteristics of statins are presented in Table 2 [79,80].

The main family enzymes involved in the hepatic metabolism of statins are the cytochrome (CYT) P450 isozymes. Atorvastatin, simvastatin, and lovastatin are predominantly metabolized by the cytochrome P450 3A4 (CYP3A4) enzyme. Fluvastatin is metabolized primarily by the CYP2C9 isoenzyme. The CYP450 pathway does not significantly metabolize pravastatin, pitavastatin, and rosuvastatin. Drugs metabolized by the CYP 450 system are more likely to cause side effects, such as myopathy, due to the risk of interaction with other drugs that induce CYP450, especially the CYP3A4 isoform. A proportion of the inhibitory activity of some statins on HMG-CoA reductase is attributed to their active metabolites (Table 2). For example, the major active metabolites of atorvastatin are 2-hydroxy and 4-hydroxy-atorvastatin acids, and of simvastatin, β-hydroxy acid, and its 6′-hydroxy, 6′-hydroxymethyl and 6′-exomethylene derivatives. The main elimination route for statins is by the liver, so caution is mandatory when recommending these drugs to patients with liver disease. Another way to eliminate statins is active excretion by the kidneys. If in the case of atorvastatin, renal elimination is <5%, in the case of rosuvastatin, the renal elimination reaches up to 10%, and in the case of cerivastatin, up to 30%. Thus, the pharmacokinetic properties of rosuvastatin do not change in patients with mild or moderate hepatic impairment [80].

To obtain maximum benefits and minimal adverse reactions of statin treatment, optimal doses should be used (the right dose defined as the dose that lowers LDL-C levels under 100 mg/dL, and under 70 mg/dL in patients with very high cardiovascular risk) [74]. It is important to understand the differences between genders regarding statin metabolism. These differences can be explained by mechanisms, such as:

- In women, the CYP3A4 enzyme expression is twice as high when compared to men. Consequently, the metabolism of CYP3A4-dependent statins is faster, and their activity is lower than in men.
- In women, muscle mass is lower when compared to men; hence, they are more vulnerable to myopathy.
- In women, the percentage of fat tissue is higher when compared to men. As such, the distribution volume of lipophilic statins, such as simvastatin, is higher, and the maximum serum concentration is lower.
- In women, the glomerular filtration rate is suggested to be lower when compared to men. As such, women may be more exposed to the risk of developing adverse reactions [81,82].

In conclusion, there are differences between men and women regarding the statin metabolism and LDL-C level changes.

## 4. Benefits vs. Risks of Statin Treatments

There is a clear proof of the benefits of statin treatment in the reduction of cardiovascular morbidity/mortality by reducing the levels of LDL-C, as well as regression or stabilization of coronary atheromatous plaques [83].

The reduction of mortality was evident in the Long-Term Intervention with Pravastatin in Ischemic Disease (LIPID) study [84], which included over 9000 patients with unstable coronary heart disease (CHD). In this clinical trial, it was shown that pravastatin reduced all-cause mortality by 14% and cardiovascular mortality by 24%, with a 29% reduction in nonfatal MI. As shown by the Heart Protection Study, the survival advantage of statin treatment was independent of the baseline cholesterol level; simvastatin therapy was associated with a reduction of all-cause mortality by 13% and a reduction of cardiovascular mortality by 18%, across a wide range of initial LDL levels.

Another meta-analysis of 25 trials that included 70,000 patients with CHD found that statin therapy, on average, reduces the risk of CVD events by 25% and overall mortality by 16% [85].

A relatively recent meta-analysis was published by the Cholesterol Treatment Trialists’ (CTT) Collaboration, to provide a more detailed assessment of the benefits of statins in men and women, for both primary and secondary prevention of vascular and non-vascular outcomes [86]. Earlier meta-analyses on this subject evaluated the effect on clinical prognosis, without taking into account the baseline risk of CHD, which represents a major limitation. Women usually develop CHD at an older age than men, which makes it is extremely important to take into account the gender, as well as the individual’s risk of CHD. The main outcomes analyzed by the CTT researchers were major coronary events (MI or coronary death), major vascular events, coronary artery revascularization, stroke, site-specific cancers, and cause-specific mortality. To be sure that outcomes were determined among men and women of similar baseline risk of CHD, the authors used Cox proportional hazards models (Cox, 1972) [87], which is mainly considered a regression model, often used in investigative medical studies to assess the association between subject survival time and one or more predictive variables. This model was applied to classify patients into one of four baseline categories of 5-year risk of a major cardiovascular event, as follows: <10%, 10–20%, 20–30%, and over 30%. The study included data from 27 trials: 22 trials were statin vs. control, and 5 trials were performed with higher intensity statin therapy vs. lower intensity statin. Among all the analyzed studies, 46,675 (27%) of the 174,149 patients were females. Across all the trials, statin therapy (or higher intensity statin therapy) decreased TC and LDL-C and compared with the control (or lower intensity statin) from baseline to one year, in both men and women, by similar values. Statin therapy also reduced the risk of major vascular events by 21% for each 1.0 mmol/L reduction in LDL-C (RR 0.79, 95% CI 0.77–0.81, *p* < 0.0001), with significant decreases in both sexes. The proportional reductions in major vascular events were similar between men and women with a history of vascular disease, but the effects in patients with no history of vascular disease were higher in men (RR 0.72, 99% CI 0.66–0.80) than in women (RR 0.85, 99% CI 0.72–1.00). The population of patients without established vascular disease included patients with comorbidities (kidney disease and DM) that would place them at higher risk. After the adjustment for no sex differences, there was a similar reduction in all-cause mortality per 1 mmol/L decrease of LDL-C, namely of 10% in men (RR 0.90, 99% CI 0.86–0.95) and 9% in women (RR 0.91, 99% CI 0.84–0.99).

**Figure 1:**
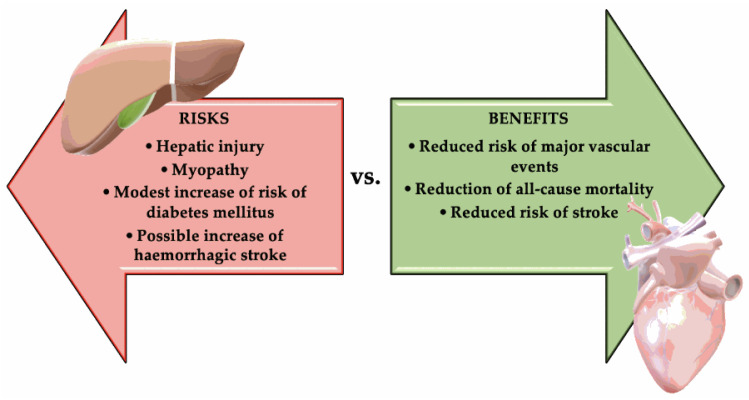
The risks vs. benefits of statins treatment.

As such, when weighing the benefits and risks of statin treatment, this scale leans in favor of the benefits. The risks, however, are not to be underestimated:

- Modest risk of developing DM (approximately 0.1% annually). In an analysis from the Justification for the Use of Statin in Prevention: An Intervention Trial Evaluating Rosuvastatin (JUPITER) trial, statin therapy accelerated the time to new-onset diabetes by only 5.4 weeks. This is a weak reason not to use statins [88].
- Muscular symptoms, such as myalgia [89]. However, the specific diagnosis of “drug-induced myopathy” was established in only 0.6% of cases [90].
- Very rarely: Clear clinically evident hepatic injury, most frequently being asymptomatic, and usually temporary elevation of aminotransferases [91].
- Possible increase of hemorrhagic stroke in a patient with a history of stroke. This risk was suggested by Stroke Prevention by Aggressive Reduction in Cholesterol Levels trial (SPARCL), but it was not confirmed by studies that used computed tomography (CT) with contrast substance [83]. The risk of hemorrhagic stroke in SPARCL correlated with prior history of hemorrhagic stroke and inadequately controlled blood pressure, not with statin or LDL-C reduction per se [92]. Figure 1 summarizes the risks vs. benefits of statins treatment.

Furthermore, until now, there is no evidence of a greater stroke risk in patients without cerebrovascular history, nor is there evidence of statin-related adverse reactions on cognitive function, significant clinical deterioration of the renal function, or risk of cataracts [83]. As for myopathy, several factors frequently associated with it have been identified (Table 3) [93,94].

A careful study to evaluate clinical indications concerning statin-induced muscle reactions looks into the most indicated phenotypes for each patient. However, as a general guideline, reviewing the properties of statins for patients with cardiovascular risk is suggested to avoid side effects, such as myopathy or the incidence of another myopathy (i.e., Steinert myotonic dystrophy or McArdle disease) and determine if increased physical activity is connected to the patient’s state and manifestations [95].

In a patient suffering from myalgia, with serum creatine kinase (CK) values <1000 IU/L, less than 5 times the superior limit of normal and showing no muscle weakness during the physical examination, the administration of statin may be advisable, while monitoring CK levels that are to be stable or normal at the next examination. When the CK level reaches a value 10 times greater than the superior limit, the physician has to evaluate the danger/advantages of giving up the drug treatment, even if the drug may be needed. As a general guideline, in a patient at high risk who previously suffered an MI, presenting myalgia with normal serum CK levels and without muscle weakness, ceasing the statin administration is not advisable [96]. In clinical practice, muscle symptoms related to statin administration are frequent and occur in about 30% of patients [96]. A recent study performed by a Canadian team presented six main directions in managing subjects intolerant to statins. Their approach requires that subjects receive statin correctly, being aware of the advantages and risks of the treatment, as well as looking into the factors that could enhance the risk of statin-associated myopathy. A careful evaluation of the laboratory and clinical data of the patient has to be carried out for every subject showing muscle symptoms during statin treatment, then using a de-challenge or a re-challenge procedure. Despite intolerance to a certain statin, determined by muscle symptoms, most subjects can tolerate another statin; as there is no proof of cardiovascular advantages of non-statin drugs used alone, this fact is of the utmost relevance [96].

As previously mentioned, one of the main factors that contributes to the discontinuation of statin treatment is myalgia. To prevent the development of muscular symptoms, it is necessary to pay attention to factors associated with this adverse reaction. As for the management of statin-induced myopathy, the following strategies are recommended:

- In a patient with myalgia, if the CK levels exceed the reference range limit by 4 times, therapy should be discontinued for 2 to 4 weeks, and after this, the statin dose should be lowered and then titrated to the tolerated dose or another statin can be used [97].
- In a patient with myalgia, if the CK levels are not elevated or small elevations are present, the therapy should be discontinued for 2 weeks. If the symptoms improve, statin therapy can be continued. If the symptoms persist, the statin must be changed; if with the new statin symptoms no longer occur, the same drug can be continued, while if symptoms reoccur, the highest tolerated statin dose should be used, by changing the statin, starting with a low dose, and considering an alternating dosage [97].

To avoid drug interactions, the US Food and Drug Administration has the following recommendations for simvastatin treatment [97]:

- Simvastatin is contraindicated with: itraconazole, ketoconazole, posaconazole, erythromycin, clarithromycin, telithromycin, human immunodeficiency virus protease inhibitors, nefazodone, gemfibrozil, cyclosporine, or danazol [97].
- Do not exceed 10 mg simvastatin daily when taking verapamil or diltiazem [97].
- Do not exceed 20 mg simvastatin daily when taking amlodipine, amiodarone, or ranolazine [97].
- Avoid drinking large quantities of grapefruit juice [94,98].

## 5. Statin Treatment in Fertile Women

The effects of contraceptive drugs on blood lipids should be considered (Table 4). The current recommendation is that women with familial hypercholesterolemia choose a contraceptive drug with reduced androgenic activity [99,100,101].

The teratogenic effects of statins on the fetus have been evaluated. Documentation regarding contraceptive methods is necessary before statin therapy initiation in fertile women. Moreover, repeatedly informing the patient regarding the importance of avoiding pregnancy during the treatment is necessary, as well as the importance of stopping the treatment 4–6 weeks before ceasing contraception. However, available data are contradictory, and the teratogenic risk associated with the use of statins is still unclear; until further consistent data, statins remain contraindicated in pregnant women [102,103,104,105].

## 6. Statin Treatment in Postmenopausal Women

Menopause is a life period that directly impacts cardiovascular risk. It is estimated that 46 million women in the US will reach menopause by 2020 [106]. Cholesterol values increase during this period until they reach a plateau. This suggests the necessity of adjusting statin treatment in menopausal women, as these patients have higher cholesterol levels [107]. 

Age is an independent cardiovascular risk factor. In total, 70% of the population aged 65 to 80 years suffer from CVDs, and this percentage rises to 85% for those aged 80 and above [102,103]. From this perspective, it is necessary to adapt the treatment according to the patient’s age, because elderly patients have certain clinical features that should be considered:

- Risk of drug interactions. The therapeutic regimens of the elderly frequently include more than five drugs, including dietary supplements. As such, the risk of interaction between statin metabolism and other drugs, CYP3A4 and cytochrome P450 2C9 (CYP2C9), increases [108,109].
- Pharmacokinetic changes, from intestinal absorption to bioavailability and distribution volume. The risk of myopathy rises because of muscle atrophy associated with advanced age. By reducing the distribution volume, similar effects can be obtained, albeit with a smaller dose [110,111].
- Treatment costs for the elderly.
- Patient adherence/tolerance to the treatment.
- Hypothyroidism, which can lead to muscle symptoms that can be misinterpreted as secondary effects of statin treatment [99].

Hypothyroidism is a well-known cause of secondary dyslipidemia, and its link to atherosclerosis has been known for 125 years. Elevated circulating levels of the apolipoprotein B100 containing lipoproteins, very low-density lipoprotein, and low-density lipoprotein are the main lipid abnormalities seen in patients with hypothyroidism.

For spontaneous myopathy and also statin-induced myopathy (SIM), hypothyroidism is considered a risk factor. Suggestive clinical symptoms and signs are cramps, muscle pains, and weakness [112,113,114]. Simultaneous intake of inhibitors of hepatic cytochrome P-450 and fibrates, surgery, or serious trauma represents other factors that enhance the SIM risk [113]. Isolated hypothyroidism may very seldom cause rhabdomyolysis, with the most serious being an occasionally fatal complication of SIM. Clinical data indicate a ratio of SIM between 0.1% and 0.2% for the general population; there is no evidence for the percentage of myopathy determined by hypothyroidism. Given these facts, it is advisable to evaluate the thyroid status of subjects before administering lipid-lowering drugs, and in the case of patients under statin treatment, thyroid activity has to be evaluated when resistance to therapy or myopathic symptoms occur [115]. For patients with hypothyroidism, statin treatment can enhance the risk of rhabdomyolysis and myopathy. The literature presents various cases of patients with hypothyroidism under statin treatment who developed rhabdomyolysis [116]. In addition to these scenarios, a PubMed literature search identified two cases of statin-induced myopathy occurring in the setting of induced hypothyroidism. Neither of the previously reported cases resulted in overt rhabdomyolysis or the need for treatment of acute kidney injury. Possible explanations for how hypothyroidism might enhance statin myopathy include decreased clearance of CK or decreased drug catabolism, resulting in higher serum statin levels [117]. 

## 7. Conclusions

Currently, most physicians rely only on lipid-lowering molecules to treat individuals with dyslipidemias. We emphasize that lifestyle modifications should be paramount in all cases, in addition to standard therapy. Combined measures aiming at all modifiable risk factors would minimize the risk of CHD, MI, HF, and death. It is axiomatic that concomitant treatment of two or more risk factors should add extra benefits in preventing atherosclerotic vascular events.

Data from the literature show that, independent of gender, the use of statins leads to a decrease in LDL-C, regression or stabilization of atherosclerotic plaques, and a reduction in cardiovascular morbidity and mortality [61]. However, there are some differences between women and men concerning the metabolism of statins; individualization of treatment to obtain the aforementioned benefits is therefore necessary [59,60].

Optimization of statin treatment in women requires adjusting the doses by renal function and muscle symptoms. Moreover, informing the patient regularly about the risks and benefits is necessary to improve treatment adherence. As for elderly women, focusing on drug interactions, simplifying the treatment, and evaluating the adherence to treatment is of utmost importance [118,119,120]. Statin treatment in women will be initiated unless cholesterol levels do not reach target values after 3 to 6 months of lifestyle changes, including an appropriate diet, reduced alcohol consumption to less than seven drinks per week, and physical exercise. Therapy must be individualized according to the preferences and wishes of female patients, keeping in mind the pharmacokinetic differences with men. Further studies are required, for a better understanding of CVDs and treatment particularities in women and to identify additional cardiovascular risk factors.

## Figures and Tables

**Table 1:** Cardiovascular risk factors unique to women [64].

Cardiovascular Risk Factors Unique to Women
Early menarche Premenstrual syndrome Menopause Hysterectomy Combined estrogen-progestin oral contraceptives Polycystic ovary syndrome Pregnancy complications (hypertension and diabetes mellitus, spontaneous pregnancy loss, placental abruption, and others)

**Table 2:** Pharmacokinetic properties of statins [80].

Statin	Solubility	Effect of Food	CYP450 Metabolism and Isoenzyme	Active Metabolites	Renal Excretion (%)	Elimination Half-Life (h)
**Fungal-derived statin**						
**Lovastatin**	Lp	BA increased	3A4	yes	10	3
**Pravastatin**	Hp	BA decreased	–	no	20	1.8
**Simvastatin**	Lp	no effect	3A4	yes	13	2
**Fully synthetic compounds**						
**Atorvastatin**	Lp	BA decreased	3A4	yes	<5	14
**Cerivastatin**	Lp	no effect	3A4, 2C8	yes	30	2.5
**Fluvastatin**	Lp	BA decreased	2C9	no	6	1.2
**Pitavastatin**	Lp	not available	limited	minor	not available	11
**Rosuvastatin**	Hp	no effect	limited	minor	10	19

Lp–Lipophilic; Hp–Hydrophilic; BA–Bioavailability; 3A4, 2C8, 2C9–cytochrome P450 (CYP450) enzymes.

**Table 3:** General factors associated with the highest risk of myopathy vs. specific factors for statin-induced myopathy [93].

General Factors Associated with the Highest Risk of Myopathy	Specific Factors for Statin-Induced Myopathy
Elderly, especially >80-year old Female sex Low body mass index Hypothyroidism Renal or hepatic impairment Diabetes mellitus Recent surgery	Advanced age (>80-year old) The genetic risk factor for simvastatin myopathy (SLCO1B1 rs4149056) Hypothyroidism Severe chronic kidney disease Impaired liver function Perioperative period
Interacting medications (macrolide antibiotics, fibrates, cyclosporine, amiodarone, verapamil, antifungals, protease inhibitors)	Alcohol abuse Consumption of large quantities of grapefruit juice Interacting medications

**Table 4:** Effects of hormonal contraceptives on lipid levels.

Contraceptive	LDL-C	HDL-C	Triglycerides *
Combination estrogen/progestin pills	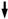	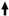	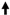
Estrogen component	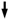	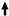	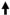
Progestin component	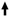	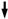	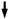
Transdermal patch	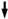	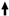	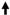
Vaginal ring	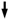	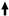	No change
Depot medroxyprogesterone acetate	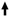	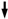	No change

* A modest increase in triglycerides does not increase the risk of atherogenesis.
